# Are annoyance scores based on sound pressure levels suitable for snoring assessment in the home environment?

**DOI:** 10.1007/s11325-020-02108-y

**Published:** 2020-05-27

**Authors:** René Fischer, Franziska Unverdorben, Thomas S. Kuehnel, Veronika Vielsmeier, Gerrit Spanier, Steven C. Marcrum, Christian Rohrmeier

**Affiliations:** 1 Department of Otorhinolaryngology, University of Regensburg, 93042 Regensburg, Germany; 2 Department of Cranio-Maxillofacial Surgery, University of Regensburg, 93042 Regensburg, Germany; 3 Faculty of Medicine, University of Regensburg, 93042 Regensburg, Germany; 4 ENT Medical Office, Bahnhofstr. 19, 94315 Straubing, Germany

**Keywords:** Snoring, Assessment, Annoyance, Psychoacoustic snore score, Sound pressure level

## Abstract

**Purpose:**

An objective statement about the annoyance of snoring can be made with the Psychoacoustic Snore Score (PSS). The PSS was developed based on subjective assessments and is strongly influenced by observed sound pressure levels. Robustness against day-to-day interfering noises is a fundamental requirement for use at home. This study investigated whether or not the PSS is suitable for use in the home environment.

**Methods:**

Thirty-six interfering noises, which commonly occur at night, were played in the acoustic laboratory in parallel with 5 snoring sounds. The interfering noises were each presented at sound pressure levels ranging from 25 to 55 dB(A), resulting in 3255 distinct recordings. Annoyance was then assessed using the PSS.

**Results:**

In the case of minimally annoying snoring sounds, interfering noises with a sound pressure level of 25 dB(A) caused significant PSS changes from 40 to 55 dB(A) for annoying snoring sounds. If the interfering noise was another snoring sound, the PSS was more robust depending on the sound pressure level of the interfering noise up to 10 dB(A). Steady (no-peak) interfering noises influenced the PSS more strongly than peak noises.

**Conclusions:**

The PSS is significantly distorted by quiet interfering noises. Its meaningfulness therefore depends strongly on the acoustic environment. It may therefore be assumed that scores dependent on sound pressure level are suitable for measurements when there is minimal ambient noise, as in the sleep laboratory. However, for measurements where noise is incalculable, as in the home environment, interfering noises may distort the results.

## Introduction

Snoring is commonly described as a nuisance, especially by bed partners. As a consequence, problems initiating and maintaining sleep and resulting daytime tiredness, separate bedrooms, and tension in the relationship are not uncommon [1–4]. It is therefore unsurprising that a partner’s stress is often given as the primary reason why habitual snorers wish to obtain treatment [5, 6].

The physician’s task is to assess snoring severity, determine the optimal treatment plan in light of the given risk-benefit relationship, and monitor treatment outcome. One difficulty is that there are often discrepancies between the subjective reports of the snorer and bed partner, as well as between subjective and objective measures [7–10].

Indices calculated from acoustic parameters are commonly used to assess snoring frequency and severity. These include, for example, the Snoring Index (SI), which denotes the number of snoring sounds per hour or the percentage or absolute snoring time [7, 11]. For this purpose, sounds above a specified decibel threshold are rated as snoring.

Other snoring scores use psychoacoustic parameters to provide a more valid and, above all, objective assessment of annoyance for the bed partner [12]. One example is the Berlin Snore Score (BSS) developed by Caffier et al. in 2007. The BSS is a logarithmic formula which is based primarily on different percentiles of sound pressure levels [13]. Another option is the Psychoacoustic Snore Score (PSS), which was developed using regression analysis and was based on the subjective assessments of different snoring sounds by study subjects [14]. The PSS is calculated from psychoacoustic parameters assessing both loudness and roughness. The aim of the PSS is to make an objective, measurable statement about the “subjective” annoyance of snoring sounds. Depending on the height of the PSS, snoring sounds can be divided into different levels of annoyance. It can be assumed that snoring sounds are perceived as very annoying at PSS values above 90, as moderately annoying at values around 60, and as hardly annoying at values below 40 [14].

Common to all these measures is the use of either sound pressure level or parameters which strongly correlate with sound pressure level. It is therefore of great interest to determine whether interfering noises, such as traffic noise or potential snoring by the bed partner, could have a considerable influence on these indices and scores. The effect of sound pressure level may be particularly relevant when acoustic measurements are not obtained in the sleep laboratory, but rather in a patient’s home. Compared to measurements in a sleep laboratory, snoring measurements at home offer the advantage of significantly lower costs and the possibility of easier and more frequent (e.g., postoperative) snoring controls. A measurement would be possible with a microphone alone without any further technical effort on the patient (no cabling), so that natural sleep would not be affected. This would even be possible via an app in the smartphone, for example.

The aim of the present study was therefore to establish whether the PSS, as an example of scores that are related to the sound pressure level, is suitable for the home environment. Robustness against day-to-day interfering noises in the home is a fundamental requirement for this setting.

## Materials and methods

### Acoustic material

In order to be able to make a statement about a broad spectrum of snoring sounds, 5 usual snoring sounds of varying annoyance (PSS 12, 27, 51, 59, and 91) were selected. These sounds were from 5 snorers (4 male; mean age 44.2 years; 28–63 years), all of whom had been examined by polysomnography. The apnea-hypopnea indices of the snorers were 0.9, 1.6, 5.8, 19, and 24, each per hour. Furthermore, 36 different night-time interfering noises were used for the study. Characteristic and generally familiar sounds that commonly occur, especially at night were used as the interfering noises (Table 1). These largely originate from the areas of traffic noise, nature and the environment, household and social noise, as well as sounds that can occur in the bedroom (sources: Widder Musik Hamburg, Rundfunk, [15]). With the aid of the psychoacoustic parameter of fluctuation strength (*F*_max_ *in vacil*), these interfering noises were divided into steady (no-peak; < 90 vacil) and non-steady (peak; > 90 vacil) sounds (Table 1). The degree of fluctuation strength is a measure of the subjectively perceived fluctuation in loudness; it is not necessarily related to annoyance. All the sequences were cut to a length of 10 s; in some instances, the sounds therefore had to be repeated frequently. The snoring sequences always contained exactly two snoring sounds in this time period. The interfering noises were amplified to a specified maximum sound pressure level (25, 30, 35, 40, 45, 55 dB(A)). The Adobe Audition 3.0 software (Adobe, San Jose, USA) was used. The recordings were stored in WAV format (Windows PCM) with 48,000 Hz, 32-bit as mono-signals.

**Table 1:** Interfering noises

Steady (no-peak) interfering noises			Non-steady (peak) interfering noises		
	*F*_max_	PSS		*F*_max_	PSS
Stormy weather	1.5	79.0	Baby	90.2	41.3
Airplane	8.5	72.7	Motorbike	104.9	70.8
Washing machine	8.7	78.1	Breathing	105.1	66.0
Rainfall	13.1	81.2	TV	105.3	74.7
Sirens	13.4	41.2	Scratching	109.6	68.8
Street cleaning	16.2	75.7	Lightning strike	114.6	40.1
Long-distance train	21.2	67.1	Bed covers	117.7	70.3
Garbage collection	24.7	73.1	Floorboards	129.2	44.1
Bell ringing	25.8	65.9	Coughing	138.1	50.3
Car	27.7	61.3	Dog barking	138.7	44.2
Party	31.5	52.8	Alarm clock	145.9	64.8
Helicopter	31.7	67.5	Vibrating cell phone	165.8	55.8
Birdsong	40.1	38.9	Car horn	167.1	50.2
Tram	46.5	59.2	Heating thermostats	167.7	45.5
Buzzing insects	48.7	72.9	Grandfather clock ticking	169.5	79.2
Snoring	52.1	73.5	Key in a lock	200.3	47.0
Telephone	54.1	32.1	Doors slamming	205.9	21.4
Stairs	67.1	75.1			
Toilet flush	76.0	68.0			

The table shows the 36 interfering noises used, sorted by their psychoacoustic fluctuation strength (*F*_max_ in vacil), and also indicates their acoustic annoyance, calculated with the PSS. For the calculation, all interfering noises were amplified to a sound pressure level of 55 dB(A). The noises were divided according to their fluctuation strength into steady (< 90 vacil) and non-steady (> 90 vacil) interfering noises. The degree of fluctuation strength is a measure of the subjectively perceived fluctuation in volume; it is not necessarily related to annoyance

### Study design

**Fig. 1:**
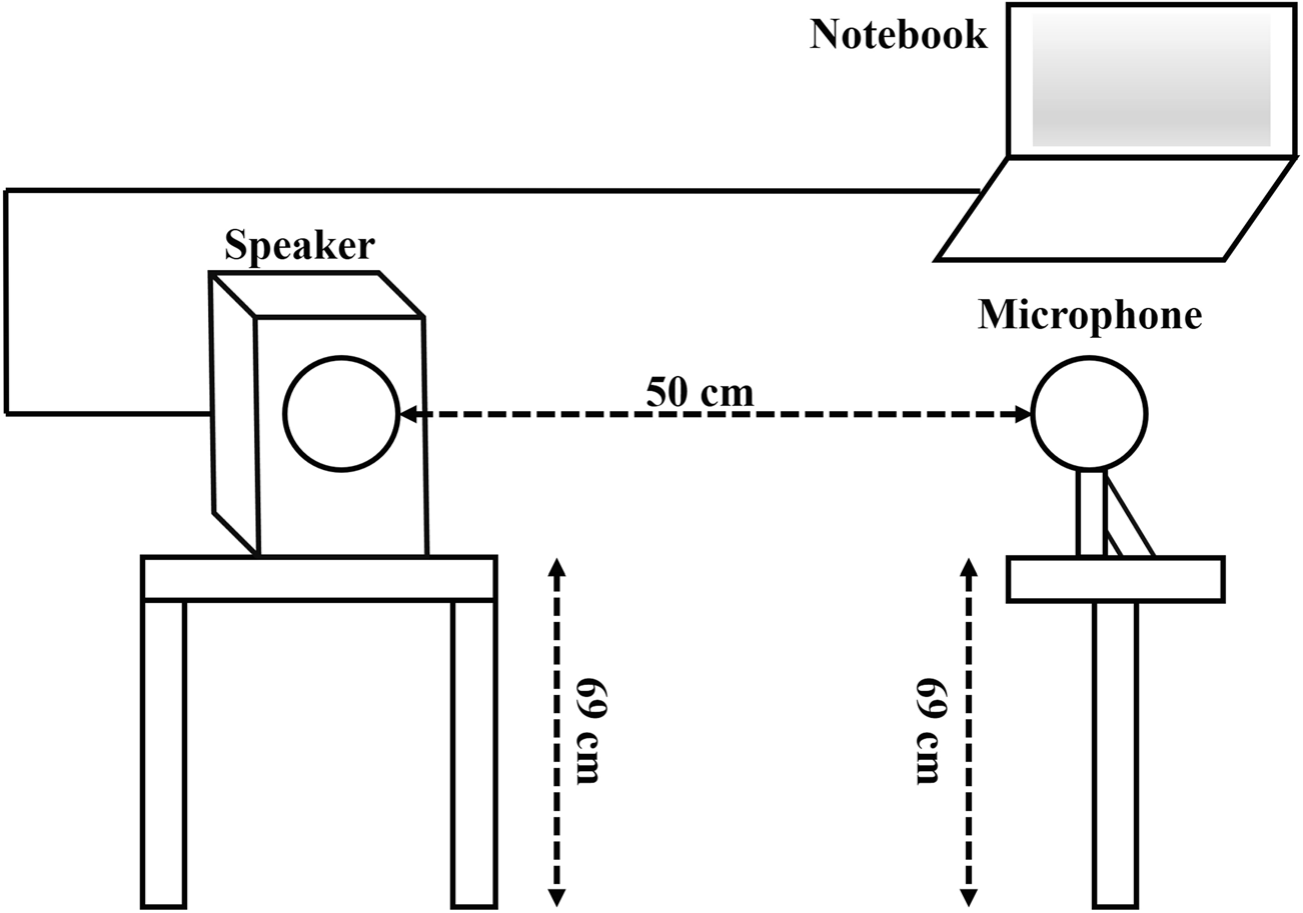
Study design. In a soundproof room, the snoring and disturbing noises were each reproduced by a loudspeaker (positioned at a height of 69 cm) and then recorded by a microphone (distance to speaker 50 cm)

The tests were performed in a soundproof room (room size 11.5 m^2^, height 2.5 m; reverberation time < 0.1 s). Each time, one snoring sequence and one interfering noise were played simultaneously three times in succession via the Adobe Audition 3.0 software (Fig. 1). The sound was produced by a loudspeaker with integrated sound card (nuPro A-20, Nubert electronic, Schwaebisch Gmuend/Germany) positioned at a height of 69 cm. Exactly 50 cm from the loudspeaker, a class 1 microphone with an audio and acoustic analyzer (XL2 with M2210, NTi Audio AG, Schaan/Liechtenstein) was placed, through which the individual playbacks were recorded. This resulted in a total of 3255 recordings (5 snoring sequences × 36 interfering noises × 6 different sound pressure levels each × 3 runs; in addition, 5 snoring sequences without interfering noises × 3 runs).

### Acoustic analyses

From the audio files recorded in WAV format, objective calculations were made with the dBSONIC Version 4.13 software (01dB-Metravib technologies, Limonest cedex/France). Sound pressure level ([dB], A-weighted) and the psychoacoustic parameters of loudness (*N* in sone), roughness (*R* in asper), and fluctuation strength (*F* in vacil) were determined. These values were used to calculate the Psychoacoustic Snore Score (PSS), which indicates the annoyance of snoring sounds [14]:

$$ \mathrm{PSS}=28.5\times \ln \frac{N_5}{\mathrm{sone}}+11.3\times \ln \frac{R_{\mathrm{mean}}}{\mathrm{asper}}-23.9 $$

where *N*_5_ is the 5th percentile of loudness and *R*_mean_ is mean roughness.

### Question, statistics

The study investigated whether interfering noises lead to significant changes to the PSS and, if so, above what sound pressure level. As data not normally distributed were present, a multifactor analysis of variance (ANOVA) with repeated measures was performed. Sphericity was tested with Mauchly’s test. As it was not present, the Greenhouse-Geisser correction was applied. A Bonferroni correction was also made.

Microsoft Excel 2016 for Windows software (Microsoft Corporation) and SPSS Statistics 24 software (IBM Corporation, Armonk/USA) were used for the statistical analysis and creating graphs. Values below 0.05 were classed as significant, values below 0.01 as highly significant.

## Results

**Fig. 2:**
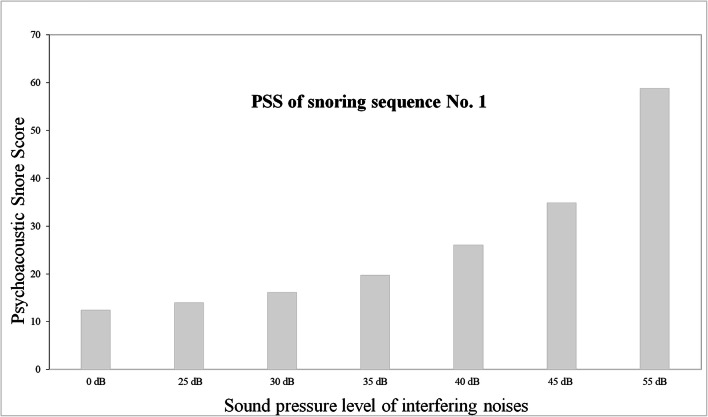
Example snoring sequence No. 1. The diagram shows how the interfering noises change the PSS of the snoring sequence No. 1 at different loudness: 0 dB corresponds to the PSS of the snoring sound alone. If the interfering noises have a loudness of 25 dB, the PSS of the snoring sound already changes significantly (*p* = 0.027, see Table 2). If the loudness of the interfering noises is even higher (30 dB and higher), the PSS increases even more, so the snoring sound is considered more annoying than it actually is. The bars indicate the average value of the PSS

**Fig. 3:**
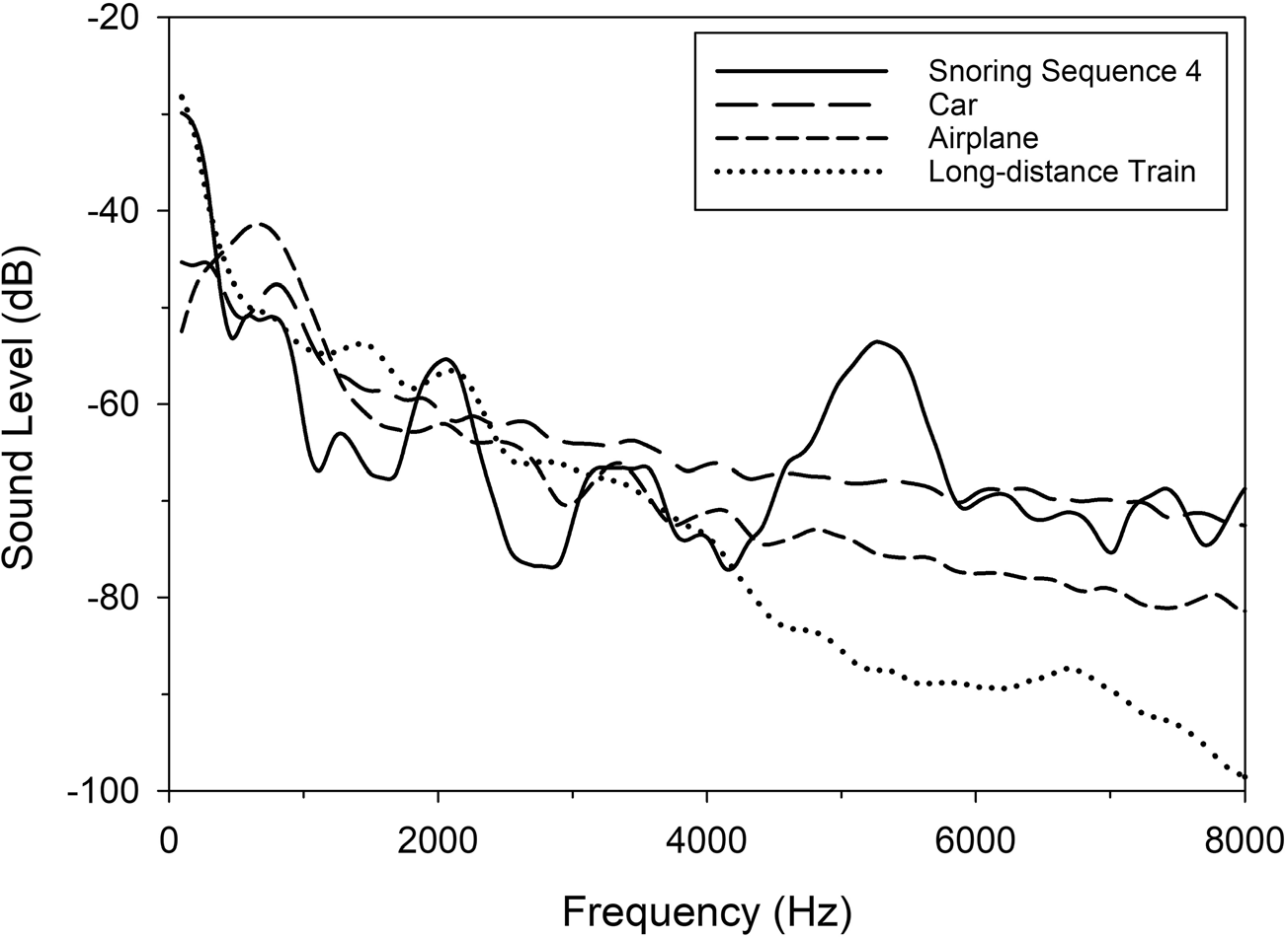
Snoring sequence No. 4 with interfering noises. A 2048-point fast-Fourier transform (FFT) was applied to snoring sequence No. 4 and the interfering noises car, airplane, and long-distance train (all with 55 dB). Results are displayed up to a frequency of 8 kHz, as signals within this frequency range demonstrate the greatest potential for impacting sleep quality. It can be seen that a very similar sound level exists in wide frequency ranges

The interfering noises led to significant changes to the PSS, but there were differences between the results for individual snoring sequences. Therefore, the results were analyzed separately for each snoring sequence. Table 2 shows the lowest loudness and the level of significance above which the interfering noises led to significant distortion of the PSS. In each case, even louder interfering noises caused highly significant distortion (*p* < 0.01), whereas quieter interfering noises did not yet lead to a significant change of the PSS. Figure 2 shows this distortion in the PSS due to the increasingly louder interfering noises, exemplary for snoring sequence No. 1. Figure 3 shows an example of a Fourier transform (FFT) of snoring sequence No. 4 with different interfering noises. It can be seen that a very similar sound level exists in wide frequency ranges.

**Table 2:** Lowest significantly distorting loudness of interfering noises. Column 1 shows the number of the snoring sequence and column 2 shows the lowest distorting loudness of interfering noises above which annoyance was significantly affected. Column 4 shows the lowest distorting loudness above which annoyance was significantly affected when the interfering noise was another snoring sound. Columns 3 and 5 each show the results of ANOVA with measurement repetition and Greenhouse Geisser correction

Sequence	Due to interfering noises	ANOVA (noises)	Due to snoring sound	ANOVA (another snoring sound)
1	25 dB (*p* = 0.027)	*F* (1.65, 23.08) = 858.8, *p* < 0.001	35 dB (*p* = 0,033)	*F* (1.40, 2.79) = 35,513.9, *p* < 0.001
2	35 dB (*p* < 0.01)	F (1.83, 25.57) = 470.5, *p* < 0.001	40 dB (*p* = 0.046)	F (1.68, 3.35) = 2927.8, *p* < 0.001
3	40 dB (*p* = 0.01)	F (1.24, 17.29) = 118.3, *p* < 0.001	40 dB (*p* = 0.044)	*F* (1.90, 3.80) = 5460.8, *p* < 0.001
4	40 dB (*p* < 0.01)	*F* (1.11, 15.59) = 191.6, *p* < 0.001	45 dB (*p* = 0.034)	*F* (1.10, 2.19) = 4298.4, *p* < 0.001
5	55 dB (*p* = 0.042)	*F* (1.17, 16.37) = 15.37, *p* < 0.001	55 dB (*p* = 0.01)	*F* (1.10, 2.20) = 399.8, *p* < 0.001

Considering only the results where the interfering noise was another snoring sound, it can be seen that for three of the five snoring sequences, a significant distortion did not occur until the sound pressure level was slightly higher (Table 2). However, this PSS distortion was only clearly pronounced with the least annoying snoring sequence no. 1.

Whether there was a difference between peak and non-peak interfering noises was viewed purely descriptively for a sound pressure level of interfering noises of 55 dB(A). The non-peak noises had a greater impact on the PSS than peak noises; the mean difference between the two groups was 7.75% (Table 3). Although the difference was more pronounced in the snoring sequences with lower PSS than those with higher PSS, overall the influence of all interfering noises was substantial. Table 4 shows exemplary, which interfering noises had the least and which the greatest influence.

**Table 3:** Difference between no-peak and peak interfering noises. The table indicates the original PSS of the 5 snoring sequences and the mean annoyance of the 5 snoring sequences while playing the interfering noises (at 55 dB) at the same time, divided into no-peak and peak noises. Column 5 indicates the percentage difference

Sequence	Original PSS	Steady (no-peak) interfering noises	Non-steady (peak) interfering noises	Difference
1	12.41	63.67	54.00	11.1%
2	27.28	62.96	54.72	9.5%
3	50.94	68.55	62.98	6.0%
4	58.52	71.41	67.21	4.4%
5	91.42	93.64	93.37	0.2%

**Table 4:** Least and most distorting interfering noises. The table shows exemplary for the snoring sequences 1 (least annoying), 3 (medium annoyance), and 5 (most annoying) the interfering noises that influence the PSS least and most (at 25 dB, 40 dB, and 55 dB respectively). Steady (no-peak) interfering noises are marked bold, non-steady (peak) interfering noises are marked in italics. It can be seen that steady interfering noises (bold) have a much stronger influence on the PSS

Least distorting interfering noises				Most distorting interfering noises		
Sequence	25 dB	40 dB	55 dB	25 dB	40 dB	55 dB
1	*Floorboards*	*Doors slamming*	*Doors slamming*	**Airplane**	**Street cleaning**	**Street cleaning**
	*Key in a lock*			**Garbage collection**	**Stormy weather**	*Grandfather clock*
	*Coughing*	*Key in a lock*	**Telephone**	**Car**	**Rainfall**	
	**Toilet flush**	**Birdsong**	*Floorboards*	**Long-distance train**	**Garbage collection**	**Stormy weather**
	*Car horn*	*Heating thermostats*	*Lightning strike*	**Bell ringing**	**Washing machine**	**Washing machine**
		*Floorboards*	**Birdsong**			**Rainfall**
3	*Motorbike*	**Birdsong**	**Telephone**	**Washing machine**	*Grandfather clock*	*Grandfather clock*
	**Birdsong**	*Doors slamming*	*Doors slamming*			
	**Party**			*Car horn*	**Garbage collection**	**Washing machine**
	*Scratching*	*Vibrating cell phone*	*Dog barking*	**Rainfall**		
			*Baby*	**Stormy weather**	**Washing machine**	**Stormy weather**
		**Party**	**Sirens**			
		*Baby*		**Bell ringing**	**Rainfall**	**Rainfall**
	*Coughing*					
					**Stormy weather**	*Coughing*
5	*Key in a lock*	*Vibrating cell phone*	*Baby*	*Bed covers*	**Rainfall**	**Toilet flush**
	*Doors slamming*		*Vibrating cell phone*	**Airplane**	**Airplane**	**Rainfall**
		*Key in a lock*		**Snoring**	**Garbage collection**	*Motorbike*
	**Toilet flush**	**Telephone**	*Car horn*	**Washing machine**		**Stairs**
	**Stormy weather**	*Doors slamming*	**Telephone**		**Stairs**	*Grandfather clock*
			*Lightning strike*	**Garbage collection**	**Washing machine**	
	*Dog barking*	*Car horn*				

## Discussion

The PSS was developed using regression analysis on the basis of subjective assessments of different snoring sounds [14]. The score is largely based on loudness, which in turn correlates strongly with the sound pressure level, especially in the case of snoring sounds [12]. Loudness itself is given in “sone,” a sound at 1 kHz with 40 dB being considered the reference. The calculation is carried out for different time intervals and critical bandwidths, using a third octave filter curve. The results are variably weighted, and then converted into specific loudness values. Formation of the integral produces the total loudness value [16].

Roughness is a second, less strongly weighted parameter in the PSS formula. The impression of roughness arises above frequency modulations of over 15 Hz and reaches its peak at modulations of around 70 Hz. The base frequencies of the noises also play a relevant role; they are especially high for values around 1 kHz [16].

The study showed that the Psychoacoustic Snore Score (PSS) can be significantly distorted even by interfering noises of only 25 dB(A) loudness. Further, 25 dB roughly equate to the loudness of quiet breath sounds, but even breath sounds can be up to 46.6 dB(A) loud [17].

Above what point the PSS was distorted depended firstly on the underlying snoring sound itself: the more annoying this was, the smaller the influence of the interfering noises. This can be explained by the strong dependence of the PSS on the loudness and thus on the sound pressure level [14]. The following everyday experience, which is certainly familiar to everyone, can serve as an example: Even a car passing at normal speed can clearly disturb a quiet conversation on the pavement, while the same car would not even be noticed next to a rock concert. Secondly, the loudness of the interfering noise played a role: the louder this was, the more probable was a significant distortion. In the example just mentioned, the car would now drive faster and thus disturb the conversation even more by its increased noise.

Overall, more steady noises (non-peak noises) had a more disruptive influence on average. This can be explained by the fact that peak noises usually contain intense, but only short-lasting impulsive maxima of the sound pressure level, while non-peak noises tend to receive a continuously “high” sound pressure level. In the above example, the continuous driving noise of a car disturbs conversation on the sidewalk more than a car horn that howls briefly.

Other snoring sounds also had a significant influence on the PSS, but it was unexpected that the PSS was slightly more robust against to these sounds, depending on the tested loudness of up to 10 dB(A). Since the PSS was developed specifically for snoring sounds, it should actually be more sensitive to the influence of other snoring sounds than to other interfering noises. This was not confirmed, but with deviations of 10 dB(A) maximum, though mainly in the quiet snoring sequences, it cannot be claimed that the PSS is really more robust with regard to a snoring bed partner.

Other snoring scores, such as the Berlin Snore Score or the widely used Snoring Index (SI), which represents the number of snoring sounds per hour, also are largely based on the sound pressure level [11, 13, 18]. According to the available results, it may therefore be expected that these indices are very susceptible to interfering noises, especially when snoring is only quiet to moderately loud. To our knowledge, this high sensitivity of sound level-based indices to interfering noises has not been described in the literature so far.

Interfering noises play a major role, especially in the home environment because the ambient acoustic conditions in the home are very non-homogeneous and incalculable. On the one hand, there is a very high round-the-clock noise burden in our society, especially caused by traffic and airplane noise, but also by neighborhood noise or noise from recreational activities [19]. Around 40 million citizens of the European Union are regularly exposed to nightly noise over 50 dB caused by traffic [19].

On the other hand, relevant sounds from the bed partner often occur in the snorer’s bedroom as well as noises that can hardly be avoided, such as the rustling of bed covers during body movements. Partner’s breath sounds and especially snoring sounds are also not a rarity. For instance, in a study on the sleep quality of women living with snorers, Blumen et al. had to exclude 7 out of 23 couples. The reason was that the women themselves snored to a relevant extent [10]. Further effects from possible interfering noises can occur, depending on the location of the bedroom, its lack of soundproofing, the existing windows, and how open the windows are.

Hence more robust techniques need to be developed for measuring annoyance in the home setting; these measurements seem to be even more representative when assessing the bed partner’s real annoyance [20]. One idea would be to switch to contact microphones, but these have serious shortcomings when measuring frequencies over 100–300 Hz [21]. With free-field microphones, measuring parameters not dependent on loudness should be used, if possible. The patient simply turning over in bed can cause differences of up to 3 dB; changes in the microphone distance and differing acoustic conditions in the room can result in further variations [18, 22, 23]. More robust parameters of this kind can be frequency-based, for instance. Herzog et al. showed that the distance or the microphone position does not play a role in this respect [21]. Special acoustic methods, such as those used for instance in speech recognition, might be useful in assessing annoyance. They are already being used successfully nowadays to distinguish breath sounds and snoring sounds and to differentiate habitual and obstructive snoring [24–29].

In summary, it can be said that annoyance values such as the PSS, which are greatly influenced by the sound pressure level, definitely deliver good results for standardized surroundings that are low in background noise, such as the sleep laboratory. This makes these methods more valuable than subjective questioning of the bed partner, for instance. However, they are not suitable for the home bedroom, especially in the presence of a bed partner, because of the high susceptibility to background noise. Acoustic parameters not based on loudness, such as those used in the field of speech recognition, might deliver more robust results in the future.

## References

[1] Ulfberg J, Carter N, Talbäck M, Edling C (2000). Adverse health effects among women living with heavy snorers. Health Care Women Int.

[2] Beninati W, Harris CD, Herold DL, Shepard JW (1999). The effect of snoring and obstructive sleep apnea on the sleep quality of bed partners. Mayo Clin Proc.

[3] Blumen MB, Quera Salva MA, Vaugier I, Leroux K, d'Ortho M-P, Barbot F, Chabolle F, Lofaso F (2012). Is snoring intensity responsible for the sleep partner's poor quality of sleep?. Sleep Breath.

[4] Virkkula P, Bachour A, Hytönen M, Malmberg H, Salmi T, Maasilta P (2005). Patient- and bed partner-reported symptoms, smoking, and nasal resistance in sleep-disordered breathing. Chest.

[5] McArdle N, Kingshott R, Engleman HM, Mackay TW, Douglas NJ (2001). Partners of patients with sleep apnoea/hypopnoea syndrome: effect of CPAP treatment on sleep quality and quality of life. Thorax.

[6] Parish JM, Lyng PJ (2003). Quality of life in bed partners of patients with obstructive sleep apnea or hypopnea after treatment with continuous positive airway pressure. Chest.

[7] Hoffstein V (2007). Review of oral appliances for treatment of sleep-disordered breathing. Sleep Breath.

[8] Miljeteig H, Mateika S, Haight JS, Cole P, Hoffstein V (1994). Subjective and objective assessment of uvulopalatopharyngoplasty for treatment of snoring and obstructive sleep apnea. Am J Respir Crit Care Med.

[9] Wiggins CL, Schmidt-Nowara WW, Coultas DB, Samet JM (1990). Comparison of self- and spouse reports of snoring and other symptoms associated with sleep apnea syndrome. Sleep.

[10] Blumen M, Quera Salva MA, d'Ortho M-P, Leroux K, Audibert P, Fermanian C, Chabolle F, Lofaso F (2009). Effect of sleeping alone on sleep quality in female bed partners of snorers. Eur Respir J.

[11] Hoffstein V, Mateika S, Anderson D (1994). Snoring: is it in the ear of the beholder?. Sleep.

[12] Rohrmeier C, Herzog M, Haubner F, Kuehnel TS (2012). The annoyance of snoring and psychoacoustic parameters: a step towards an objective measurement. Eur Arch Otorhinolaryngol.

[13] Caffier PP, Berl JC, Muggli A, Reinhardt A, Jakob A, Möser M, Fietze I, Scherer H, Hölzl M (2007). Snoring noise pollution--the need for objective quantification of annoyance, regulatory guidelines and mandatory therapy for snoring. Physiol Meas.

[14] Fischer R, Kuehnel TS, Merz A-K, Ettl T, Herzog M, Rohrmeier C (2016). Calculating annoyance: an option to proof efficacy in ENT treatment of snoring?. Eur Arch Otorhinolaryngol.

[15] Norman-Haignere S, Kanwisher NG, McDermott JH (2015). Distinct cortical pathways for music and speech revealed by hypothesis-free voxel decomposition. Neuron.

[16] Fastl H, Zwicker E (2007). Psychoacoustics: facts and models.

[17] Rohrmeier C, Herzog M, Ettl T, Kuehnel TS (2014). Distinguishing snoring sounds from breath sounds: a straightforward matter?. Sleep Breath.

[18] Dalmasso F, Prota R (1996). Snoring: analysis, measurement, clinical implications and applications. Eur Respir J.

[19] Lekaviciute J, Argalasova-Sobotova L (2013). Environmental noise and annoyance in adults: research in central, eastern and South-Eastern Europe and newly independent states. Noise Health.

[20] Sériès F, Marc I, Atton L (1993). Comparison of snoring measured at home and during polysomnographic studies. Chest.

[21] Herzog M, Kühnel T, Bremert T, Herzog B, Hosemann W, Kaftan H (2009). The impact of the microphone position on the frequency analysis of snoring sounds. Eur Arch Otorhinolaryngol.

[22] Wilson K, Stoohs RA, Mulrooney TF, Johnson LJ, Guilleminault C, Huang Z (1999). The snoring spectrum: acoustic assessment of snoring sound intensity in 1,139 individuals undergoing polysomnography. Chest.

[23] Azarbarzin A, Moussavi Z (2013). Intra-subject variability of snoring sounds in relation to body position, sleep stage, and blood oxygen level. Med Biol Eng Comput.

[24] Dafna E, Tarasiuk A, Zigel Y (2013). Automatic detection of whole night snoring events using non-contact microphone. PLoS One.

[25] Ng AK, Koh TS, Baey E, Puvanendran K (2009). Role of upper airway dimensions in snore production: acoustical and perceptual findings. Ann Biomed Eng.

[26] Fiz JA, Abad J, Jané R, Riera M, Mañanas MA, Caminal P, Rodenstein D, Morera J (1996). Acoustic analysis of snoring sound in patients with simple snoring and obstructive sleep apnoea. Eur Respir J.

[27] Duckitt WD, Tuomi SK, Niesler TR (2006). Automatic detection, segmentation and assessment of snoring from ambient acoustic data. Physiol Meas.

[28] Cavusoglu M, Kamasak M, Erogul O, Ciloglu T, Serinagaoglu Y, Akcam T (2007). An efficient method for snore/nonsnore classification of sleep sounds. Physiol Meas.

[29] Abeyratne UR, Wakwella AS, Hukins C (2005). Pitch jump probability measures for the analysis of snoring sounds in apnea. Physiol Meas.

